# Subtractive genomic approach to uncover novel drug targets in *Salmonella typhimurium* and computational screening of food-based polyphenols as inhibitors

**DOI:** 10.3389/fbinf.2025.1695217

**Published:** 2025-12-03

**Authors:** Mohammed Naveez Valathoor, Subhashree Venugopal, Anand Prem Rajan

**Affiliations:** 1 Department of Bio Medical Sciences, School of Bio Sciences and Technology, Vellore Institute of Technology, Vellore, Tamil Nadu, India; 2 Department of Integrative Biology, School of Bio Sciences and Technology, Vellore Institute of Technology, Vellore, Tamil Nadu, India

**Keywords:** subtractive genome, *Salmonella typhimurium*, food-based polyphenols, molecular docking, molecular dynamic simulation

## Abstract

**Introduction:**

The rise of multidrug-resistant *Salmonella typhimurium* is a severe public health threat that renders conventional antibiotics ineffective. This study employed a computational strategy to identify a novel drug target in *S. typhimurium* and screen food-based polyphenols as potential inhibitors.

**Methods:**

A subtractive genomics approach was used to identify essential, pathogen-specific proteins. A lead target was prioritized based on its druggability, localization, and network interactions. The target’s 3D structure was then modeled for molecular docking, molecular dynamics (MD) simulations, and binding free energy calculations with a polyphenol library.

**Results:**

The screening identified UDP-N-acetylglucosamine transferase (MurG) as a promising and previously unexplored drug target. The polyphenol 6-prenylnaringenin showed a superior binding affinity for MurG compared to the antibiotic ciprofloxacin. Subsequent MD simulations and binding free energy calculations confirmed that the MurG-6-prenylnaringenin complex was significantly more stable.

**Conclusion:**

This study validates MurG as a druggable target in *S. typhimurium* and identifies 6-prenylnaringenin as a potent inhibitor. With computational metrics superior to ciprofloxacin, 6-prenylnaringenin is a promising lead compound for developing new anti-Salmonella therapeutics. Future experimental validation is required to confirm these *in silico* findings.

## Introduction

The global rise of antimicrobial and multidrug resistance in microbial pathogens is a significant public health concern. This escalating threat is largely attributed to the widespread misuse of antibiotics, including their application in poultry and cattle feeds. Furthermore, the improper administration of antibiotics in humans, such as taking incorrect dosages obtained through over-the-counter channels, is a major contributing factor to the proliferation of resistance (Alenazy, 2022; Lipsitch and Samore, 2002). *S. typhimurium* is a pathogen that primarily affects livestock, such as cattle and poultry. Although often host-specific, it can be transmitted to humans through a process known as zoonotic transmission. In humans, the infection typically manifests as an acute gastrointestinal illness with symptoms including fever, abdominal pain, diarrhea, nausea, and vomiting. In more severe cases, the infection can progress to invasive conditions like bacteremia, sepsis, osteomyelitis, reactive arthritis, and meningitis. The global public health burden of non-typhoidal *Salmonella* is significant, with estimates indicating approximately 93.8 million infections and 155,000 deaths occurring annually (Chen et al., 2013; Eng et al., 2015; Majowicz et al., 2010; Szilagyi et al., 1985; Zha et al., 2019). The bacterium has developed resistance to contemporary antibiotics through several sophisticated molecular mechanisms.

Enzymatic deactivation: Bacteria produce enzymes, such as β-lactamases, that chemically degrade or modify antibiotics, rendering them inactive before they can reach their cellular target (Edoo et al., 2017).

Target Site Modification: Mutations in the bacterial proteins that antibiotics target, like DNA gyrase and topoisomerase IV, alter the drug-binding site. This change prevents the antibiotic from binding effectively, neutralizing its therapeutic effect (Mohammed et al., 2021).

Reduced membrane permeability: The pathogen can alter the protein channels (porins) in its outer membrane, narrowing the passage and restricting the entry of antibiotic molecules into the cell (Gopikrishnan & George Priya Doss, 2023).

Active efflux pumps: Bacteria utilize specialized membrane proteins that function as pumps. These pumps actively recognize and expel antibiotics from the cell before they can accumulate to a toxic concentration (Alenazy, 2022).

Horizontal gene transfer: Resistance genes, often located on mobile genetic elements like plasmids, are transferred between bacteria. This process allows for the rapid dissemination of resistance traits throughout a bacterial population (Wise, 1999).

The proliferation of these resistance mechanisms creates a significant therapeutic challenge, as the efficacy of conventional antibiotics continues to decline. Traditional drug discovery pipelines are poorly equipped to keep pace with this rapid evolutionary arms race, as they are both time-consuming and resource-intensive. Consequently, there is an urgent need for innovative and efficient strategies capable of identifying novel therapeutic targets that can circumvent existing resistance mechanisms (Davis et al., 2013; Huang et al., 2022; Jadimurthy et al., 2023; Vaou et al., 2022). This study addresses this gap by employing a two-stage computational approach. First, subtractive genomics is applied to rationally identify essential, pathogen-specific proteins. This ensures that potential targets are absent in the human host, a critical feature for minimizing off-target toxicity. Second, this target identification is paired with the computational screening of food-based polyphenols, a class of compounds noted for their favorable safety profiles. The novelty of this work lies in the seamless integration of these methods into a streamlined discovery framework, proceeding from a novel, low-toxicity target to a promising, naturally-derived lead compound against drug-resistant *S. typhimurium* (Ahammad et al., 2024; Attar, 2024; Sarker et al., 2023).

Polyphenols are a diverse class of plant-derived compounds (phytocompounds) recognized for their extensive therapeutic potential and versatile bioactivities, with numerous studies establishing their efficacy as potent antibacterial, antifungal, antioxidant, and anti-inflammatory agents. These multifaceted properties contribute significantly to human health, positioning polyphenols as key molecules in preventive medicine. Consequently, ongoing research continues to highlight their promise as lead compounds for the development of novel therapeutic strategies against a wide range of disorders. In the search for novel therapeutic agents to combat antimicrobial resistance, natural compounds present a promising avenue. Among these, food-based polyphenols have emerged as particularly strong candidates due to their versatile bioactivities. This diverse class of phytocompounds has a well-documented record of potent antibacterial activity against a wide range of pathogens. Given their established safety and therapeutic potential, this study will focus on leveraging these natural molecules. Therefore, the primary aim of this research is to computationally screen and evaluate specific food-based polyphenols for their ability to inhibit the newly identified drug target in *S. typhimurium*, thereby laying the groundwork for a novel therapeutic strategy (Buchmann et al., 2023; Elmaidomy et al., 2022; Joshi et al., 2021; Othman et al., 2019; Rabaan et al., 2023; Rothwell et al., 2013; Tan et al., 2022; Zhong et al., 2023).

In response to the urgent need for novel therapeutics against *S. typhimurium*, a comprehensive subtractive genomics pipeline was employed to identify essential, pathogen-specific proteins as potential drug targets. Integration of this approach with the screening of bioactive, food-derived polyphenols provides a rational strategy for the discovery of novel inhibitory compounds with therapeutic potential. Systematic identification and evaluation of such targets and inhibitors were conducted through integrated computational analyses, establishing a foundation for subsequent experimental validation.

## Materials and methods

**FIGURE 1:**
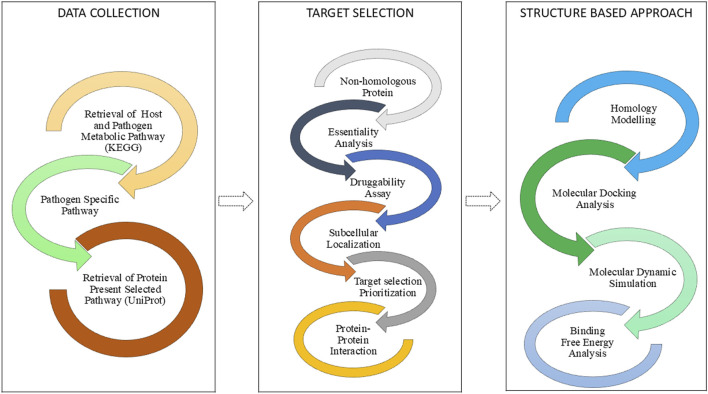
Workflow illustrating the computational pipeline for identification and evaluation of novel drug targets in *Salmonella typhimurium*.

The computational workflow of this investigation was organized into three sequential stages, as illustrated (Figure 1). The initial stage involved Data Collection, where the proteomes of the host and pathogen were retrieved. This was followed by the Subtractive Genomic Approach, a systematic filtering pipeline used to identify essential, non-homologous, and druggable protein targets unique to the pathogen. The final Structure-Based Approach involved homology modeling of the prioritized target, followed by molecular docking, molecular dynamics simulations, and binding free energy calculations to evaluate its interaction with potential inhibitors.

## Data retrieval

### Metabolic pathway analysis

To identify pathogen specific metabolic pathways, the complete pathway maps for the host (*Homo sapiens*, hsa01100) and the pathogen (*S. typhimurium*, stm01100) were obtained from the Kyoto Encyclopaedia of Genes and Genomes (KEGG) database (https://www.genome.jp/kegg/pathway.html) (accessed on May 2025). A comparative analysis was performed to isolate the metabolic pathways that are unique to the pathogen. The proteins associated with these pathogen-specific pathways were then identified, and their corresponding sequences were retrieved from the UniProt database (https://www.uniprot.org/) (accessed on June 2025). The reference proteome used for *S. typhimurium* in this study was accessed under the accession number UP000001014 (Damte et al., 2013).

### Non-homologous protein identification

To identify proteins unique to the pathogen, the retrieved protein set was subjected to a BLASTp (Protein Basic Local Alignment Search Tool) analysis against the *Homo sapiens* reference proteome in the NCBI database (https://blast.ncbi.nlm.nih.gov/Blast.cgi?PAGE=Proteins&PROGRAM=blastp&BLAST_PROGRAMS=blastp&PAGE_TYPE=BlastSearch&BLAST_SPEC=blast2seq) (accessed on June 2025). A stringent E-value threshold of 10^−4^ was applied to determine homology. Proteins showing significant similarity to human proteins were filtered out and discarded from the dataset. The remaining non-homologous proteins were then selected for the subsequent essentiality analysis (Damte et al., 2013).

### Essentiality analysis

Essential genes are those that are indispensable for an organism’s survival, as they play a critical role in fundamental cellular processes. To identify these crucial genes in *S. typhimurium*, the Database of Essential Genes (DEG) (http://origin.tubic.org/deg/public/index.php) (accessed on June 2025) was systematically queried. The database was navigated to the bacteria-specific section and searched using the keyword “*Salmonella typhimurium* LT2”. The resulting list of genes, confirmed as essential for this pathogen, was then selected for the subsequent stages of analysis (Luo et al., 2021; Subramani and Venugopal, 2025).

### Druggability assay

To evaluate the druggable potential of the candidate proteins, their sequences were compared against known therapeutic targets housed in the DrugBank database (https://go.drugbank.com/) (accessed on June 2025). This was performed using a BLASTp analysis with an E-value threshold of 10^−3^. Proteins that showed significant sequence similarity to established drug targets were considered “druggable” and were selected for further investigation (Subramani and Venugopal, 2025).

### Analysis of cellular localization

To determine the subcellular localization of the candidate proteins, their sequences were submitted to the PSORTb server 3.0.3 (https://psort.org/psortb/) (accessed on June 2025). This analysis predicts the specific location of each protein within the bacterial cell, such as the cytoplasm, cytoplasmic membrane, cell wall, or extracellular space. This information is critical for target prioritization, as a protein’s location helps determine its potential as either a therapeutic drug target (often internal) or a vaccine candidate (typically surface-exposed) (Sarker et al., 2023).

### Protein network analysis

To evaluate the functional relationships between the candidate proteins, a protein-protein interaction (PPI) network analysis was performed using the STRING database version 12.0 (https://string-db.org/) (accessed on June 2025). The purpose of this analysis was to identify which proteins serve as central hubs with a high degree of connectivity to other proteins. From this analysis, the final novel target was selected based on its strong functional associations within the network, indicating its critical role in cellular processes (Subramani and Venugopal, 2025).

### Homology modelling

The three-dimensional (3D) structure of the target protein was generated via homology modelling using the SWISS-MODEL server (https://swissmodel.expasy.org/) (accessed on June 2025, with its primary amino acid sequence as input. The crystal structure 1F0K from the Protein Data Bank (PDB) was selected as the template for model construction, as it shared a high sequence identity of 92%. Following its generation, the stereochemical quality and accuracy of the model were rigorously validated by analyzing its ERRAT score and Ramachandran plot using the SAVES v6.1 server (https://saves.mbi.ucla.edu/) (accessed on June 2025) and by calculating its overall quality Z-score with the ProSA-web server (https://prosa.services.came.sbg.ac.at/prosa.php) (accessed on June 2025) (Subramani and Venugopal, 2025).

### Ligand and protein preparation

Food-derived polyphenols were retrieved from the Phenol-Explorer 3.6 database, which contains information on 500 polyphenols from 400 different foods (Rothwell et al., 2013). These compounds were first evaluated for drug-likeness properties using three established criteria: Lipinski’s rule of five (molecular weight <500 Da, lipophilicity <5, ≤5 hydrogen bond donors, ≤10 hydrogen bond acceptors) (Lipinski, 2004), Veber rule (≤10 rotatable bonds, polar surface area <140 Å^2^), and Ghose filter (molecular weight 160–480 Da, lipophilicity −0.4 to 5.6, molar refractivity 40–130, total atoms 20–70). Compounds that satisfied these criteria were selected for further analysis. The SMILES format of each selected ligand was converted into PDB format using Open Babel GUI 2.3.1 (O’Boyle et al., 2011). For docking studies, ligands were prepared in AutoDock Tools 1.5.7 by defining the root, adding hydrogen atoms and aromatic carbons, determining torsional degrees of freedom, and assigning Gasteiger charges. The processed ligands were saved in PDBQT format. Similarly, the modeled protein was prepared in AutoDock Tools 1.5.7 by removing water molecules, adding polar hydrogens, and assigning Kollman charges before saving in PDBQT format (Valathoor and Venugopal, 2024a).

### Molecular docking analysis

The prepared ligands were docked with the modelled protein using AutoDock Vina. A grid box was set with dimensions of 62 × 48 × 70 Å at 1 Å spacing, and the grid center was defined at X = −0.956 Å, Y = 4.609 Å, and Z = 8.724 Å. Binding energies were calculated using the gradient-based optimization algorithm implemented in the program. The docking algorithm explores multiple binding conformations and clusters them by similarity. The reported binding energy corresponds to the lowest-energy pose in the most populated cluster, representing the most statistically probable binding mode. Ciprofloxacin, a commercially available antibiotic, was included as a reference compound for comparison. Based on the docking results, the highest-ranking polyphenol-protein complex, along with the ciprofloxacin-protein complex, was selected for molecular dynamics simulation studies (Subramani and Venugopal, 2025; Valathoor and Venugopal, 2024b).

### Molecular dynamic simulation analysis

Molecular dynamics simulations were performed for 100 ns on the native protein and on complexes formed with 6-prenylnaringenin and ciprofloxacin using GROMACS 2024 (Van Der Spoel et al., 2005). The CHARMM27 force field was applied to prepare the protein topology in GROMACS, while ligand topologies were generated using the SwissParam server (Vanommeslaeghe et al., 2010; Zoete et al., 2011). Each system was solvated in a cubic box with NaCl, and energy minimization was carried out using the steepest descent algorithm with the Verlet cutoff scheme to remove steric clashes. The systems were equilibrated at 300 K in two phases: first under the NVT ensemble (constant number of particles, volume, and temperature), followed by the NPT ensemble (constant number of particles, pressure, and temperature). A production MD run of 100 ns was then conducted, and trajectory analyses, including RMSD, RMSF, radius of gyration (Rg), hydrogen bonding (Hb), principal component analysis (PCA), and solvent-accessible surface area (SASA), were performed using GROMACS tools. Graphs were plotted with XMGrace (Valathoor and Venugopal, 2024c).

### Molecular mechanics generalized born surface area analysis

The molecular mechanics generalized Born surface area (MMGBSA) method was applied to estimate the binding free energy (ΔG) and evaluate the strength of the protein-ligand complexes. For this analysis, the final 20 ns of the MD simulation trajectories were used, and calculations were performed with the gmx_MMPBSA analysis tool. The final energy values are reported as the mean ±standard deviation, calculated from snapshots taken across the trajectory. This provides a quantitative measure of the interaction’s stability and the statistical confidence in the calculated binding energy from snapshots taken across the trajectory. This provides a quantitative measure of the interaction’s stability and the statistical confidence in the calculated binding energy (Bello, 2024; Valdés-Tresanco et al., 2021). This approach provided quantitative insights into the relative binding strengths of 6-prenylnaringenin and ciprofloxacin with the MurG protein.

$$\Delta G_{\text{bind}}=\Delta G_{\text{complex}}-\left(\Delta G_{\text{protein}}+\Delta G_{\text{ligand}}\right)=\Delta E_{\text{mm}}+\Delta G_{\text{sol}}\;-T\Delta S=\Delta H-T\Delta S$$

$$\Delta E_{\text{mm}}=\Delta E_{\text{vdw}}+\Delta E_{\text{ele}}+\left(\Delta E_{\text{bond}}+\Delta E_{\text{angle}}+\Delta E_{\text{dihedral}}\right)$$

$$\Delta G_{\text{sol}}=\Delta G_{\text{GB}}+\Delta G_{\text{non}-\text{polar}}$$

In the equation, ΔG_bind_ stands for the binding free energy, ΔG_complex_ for the free energy complex, ΔG_protein_ for the free energy of protein, and ΔG_ligand_ for the free energy of ligand, ΔE_mm_ stands for molecular mechanics, ΔG_sol_ stands for electrostatic solvation energy calculated using the GB model, TΔS stands for the conformational entropy after ligand binding, ΔH for enthalpy, ΔE_vdw_ for Van der Waals energy, and ΔE_ele_ for electrostatic interaction.

## Results

### Metabolic pathway analysis and protein retrieval

The KEGG database catalogs 367 metabolic pathways for *Homo sapiens* (host) and 137 pathways for *S. typhimurium* (pathogen). Comparative mapping revealed 45 pathways unique to *S. typhimurium* (Table 1), comprising 620 proteins in total. The corresponding sequences were retrieved from the UniProt database for downstream analysis.

**TABLE 1:** List of unique metabolic pathway to the *Salmonella typhimurium*.

S.No	Pathway ID	Pathway name
1	stm00261	Monobactam biosynthesis
2	stm00300	Lysine biosynthesis
3	stm00332	Carbapenem biosynthesis
4	stm00361	Chlorocyclohexane and chlorobenzene degradation
5	stm00362	Benzoate degradation
6	stm00364	Fluorobenzoate degradation
7	stm00401	Novobiocin biosynthesis
8	stm00460	Cyanoamino acid metabolism
9	stm00521	Streptomycin biosynthesis
10	stm00523	Polyketide sugar unit biosynthesis
11	stm00525	Acarbose and validamycin biosynthesis
12	stm00540	Lipopolysaccharide biosynthesis
13	stm00541	O-Antigen nucleotide sugar biosynthesis
14	stm00542	O-Antigen repeat unit biosynthesis
15	stm00543	Exopolysaccharide biosynthesis
16	stm00550	Peptidoglycan biosynthesis
17	stm00552	Teichoic acid biosynthesis
18	stm00623	Toluene degradation
19	stm00625	Chloroalkane and chloroalkene degradation
20	stm00626	Naphthalene degradation
21	stm00627	Aminobenzoate degradation
22	stm00630	Glyoxylate and dicarboxylate metabolism
23	stm00633	Nitrotoluene degradation
24	stm00643	Styrene degradation
25	stm00660	C5-Branched dibasic acid metabolism
26	stm00680	Methane metabolism
27	stm00907	Pinene, camphor and geraniol degradation
28	stm00930	Caprolactam degradation
29	stm00946	Degradation of flavonoids
30	stm00999	Biosynthesis of various plant secondary metabolites
31	stm01053	Biosynthesis of siderophore group nonribosomal peptides
32	stm01054	Nonribosomal peptide structures
33	stm01110	Biosynthesis of secondary metabolites
34	stm01120	Microbial metabolism in diverse environments
35	stm01220	Degradation of aromatic compounds
36	stm01501	beta-Lactam resistance
37	stm01502	Vancomycin resistance
38	stm01503	Cationic antimicrobial peptide (CAMP) resistance
39	stm02020	Two-component system
40	stm02024	Quorum sensing
41	stm02030	Bacterial chemotaxis
42	stm02040	Flagellar assembly
43	stm02060	Phosphotransferase system (PTS)
44	stm03070	Bacterial secretion system
45	stm03258	Virion - Bacteriophage lambda

### Non-homologous proteins identification

To exclude host homologues, all retrieved proteins were screened against the human proteome using BLASTp. A total of 123 proteins showed significant similarity to human proteins and were excluded. The remaining 497 non-homologous proteins were retained for further analyses.

### Essentiality analysis

Genes essential for bacterial viability were identified by comparison with the Database of Essential Genes (DEG). Among the 497 non-homologous proteins, only 10 were found to be essential for *S. typhimurium* survival (Table 2), and these were prioritized for subsequent evaluation (Luo et al., 2021).

**TABLE 2:** List of essential genes, their location and type of target.

S.No	Gene	Protein	Subcellular localization	Target type
1	MurB	UDP-N-acetylenolpyruvoylglucosamine reductase	Cytoplasmic membrane	Drug
2	ddlB	D-alanine--D-alanine ligase B	Cytoplasmic	Drug
3	FtsI	Peptidoglycan D,D-transpeptidase FtsI	Cytoplasmic membrane	Drug
4	PstS	Phosphate-binding protein PstS	Periplasmic	Vaccine
5	LpxA	Acyl-[acyl-carrier-protein]-UDP-N-acetylglucosamine O-acyltransferase	Cytoplasmic	Drug & Vaccine
6	PhoR	Phosphate regulon sensor protein PhoR	Cytoplasmic membrane	Drug
7	MurC	UDP-N-acetylmuramate--L-alanine ligase	Cytoplasmic	Drug
8	MurD	UDP-N-acetylmuramoylalanine--D-glutamate ligase	Cytoplasmic	Drug
9	MurE	UDP-N-acetylmuramoyl-L-alanyl-D-glutamate--2,6-diaminopimelate ligase	Cytoplasmic	Drug
10	MurG	UDP-N-acetylglucosamine transferase	Cytoplasmic membrane	Drug

### Druggability analysis

The 10 essential proteins were assessed for druggability against FDA-approved drugs from the DrugBank database. All shortlisted proteins demonstrated potential drug-binding capacity, confirming their suitability as pharmacological targets (Subramani and Venugopal, 2025).

### Sub cellular localization analysis

**FIGURE 2:**
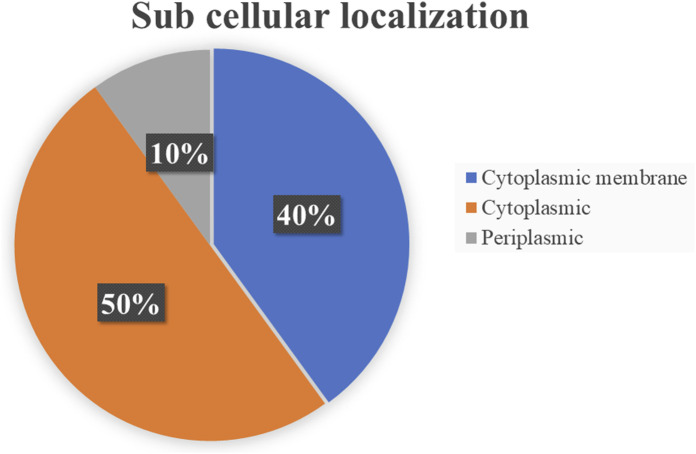
Distribution of subcellular localization of shortlisted essential proteins in *Salmonella typhimurium*.

Subcellular localization is a key determinant in distinguishing potential drug and vaccine targets. Cytoplasmic proteins are typically preferred for drug development, while membrane-associated and periplasmic proteins are prioritized for vaccine design (Alhamhoom et al., 2022). Of the 10 essential proteins, five localized to the cytoplasm, four to the cytoplasmic membrane, and one to the periplasm (Figure 2). Accordingly, while all are druggable, the periplasmic protein is particularly attractive as a vaccine candidate (Table 2).

### Protein network analysis

**FIGURE 3:**
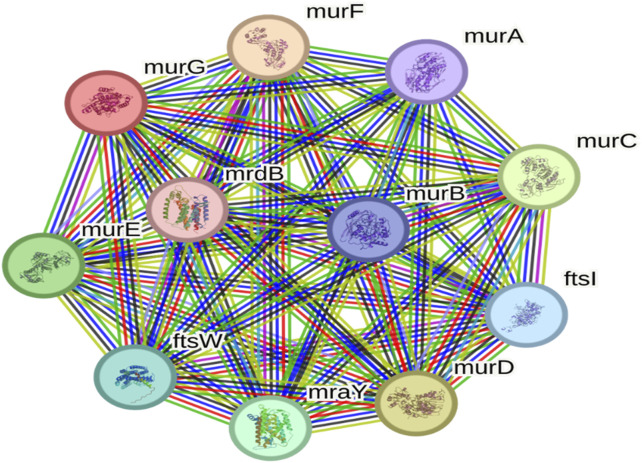
Protein-protein interaction network highlighting MurG as a central hub in *Salmonella typhimurium*.

To evaluate functional connectivity, protein-protein interaction (PPI) analysis was performed. UDP-N-acetylglucosamine transferase (MurG) emerged as a hub protein, exhibiting 11 nodes and 55 edges with an average node degree of 10 (Figure 3). Its strong interaction profile underscored MurG as a central regulator and promising therapeutic target (Subramani and Venugopal, 2025).

### UDP-N-acetylglucosamine transferase (MurG)

MurG plays a critical role in peptidoglycan biosynthesis by catalyzing the transfer of N-acetylglucosamine (GlcNAc) from UDP-GlcNAc to lipid I, generating lipid II, a key intermediate in bacterial cell wall synthesis. The UniProt ID of MurG is Q8ZRU3. It consists of 355 amino acids, with a theoretical pI of 9.71, molecular weight of 37.86 kDa, 27 negatively charged and 34 positively charged residues, an aliphatic index of 94.87, and a GRAVY score of 0.105. The instability index classified MurG as a stable protein. Given its indispensable role in cell wall construction, MurG represents a novel and high-value target for antimicrobial development (Garde et al., 2021).

### Homology modelling

**FIGURE 4:**
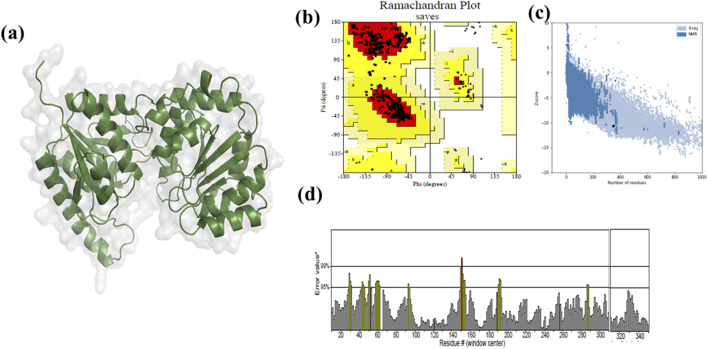
Structure of UDP-N-acetylglucosamine transferase (MurG). **(a)** Modelled protein structure **(b)** Ramachandran plot **(c)** ProsA-overall model quality **(d)** ERRAT -overall quality factor.

The three-dimensional structure of MurG was generated using Swiss-Model. Structural validation indicated high reliability, with 95.4% of residues in the most favorable Ramachandran regions, 4.6% in additionally allowed regions, an ERRAT score of 99%, and a ProSA Z-score of −10.61. These parameters confirmed the accuracy and robustness of the model for downstream analyses (Figure 4) (Sarker et al., 2023).

### Molecular docking analysis

**FIGURE 5:**
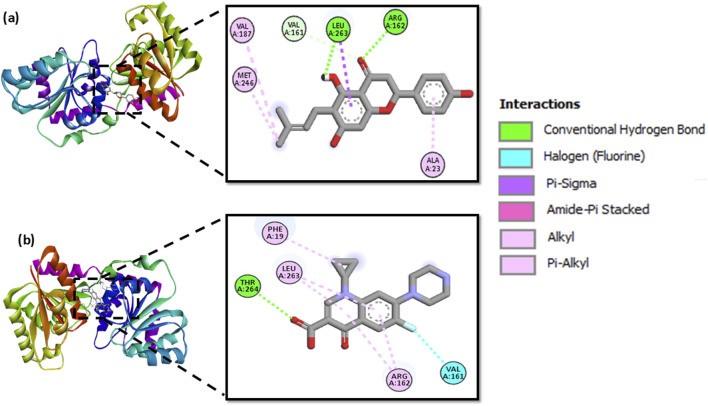
Molecular docking poses of MurG with **(a)** 6-prenylnaringenin and **(b)** ciprofloxacin, showing key interactions.

Molecular docking of MurG with 244 polyphenols produced binding affinities ranging from −8.9 to −4.9 kcal/mol. The top ten protein-ligand complexes were re-docked for validation, and 6-prenylnaringenin was identified as the most favourable candidate, with a binding affinity of −7.9 kcal/mol. This complex formed two hydrogen bonds with Arg162 and Leu263, along with additional hydrophobic contacts involving Ala23, Val161, Val187, Leu263, and Met246. By comparison, ciprofloxacin exhibited a binding affinity of −7.3 kcal/mol, forming a single hydrogen bond with Thr264 and hydrophobic interactions with Phe19, Val161, Arg162, Leu263, and Thr264. Both ligand-protein complexes (MurG-6-prenylnaringenin and MurG-ciprofloxacin) were selected for molecular dynamics simulations to further examine conformational stability (Figure 5).

### Molecular dynamic simulation analysis

**FIGURE 6:**
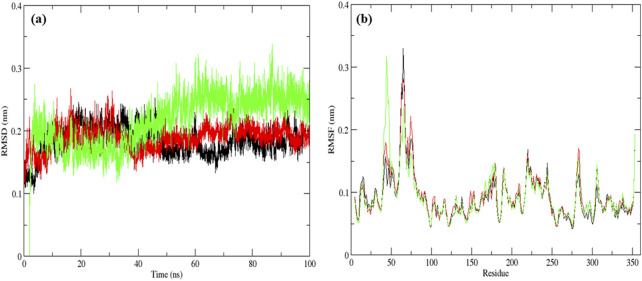
Molecular dynamic simulation results for a duration of 100 ns UDP-N-acetylglucosamine transferase (MurG) native protein (black), 6-Prenylnaringenin (red), ciprofloxacin (green) complexes **(a)** Root mean square deviation and **(b)** Root mean square fluctuation.

**FIGURE 7:**
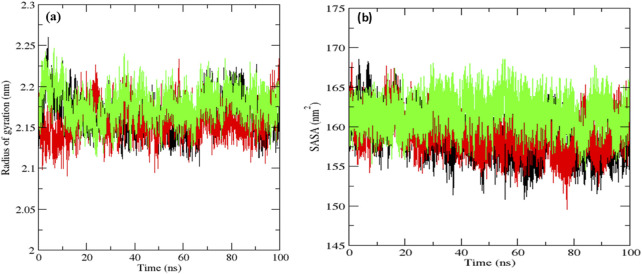
Molecular dynamic simulation results for a duration of 100 ns UDP-N-acetylglucosamine transferase (MurG) native protein (black), 6-Prenylnaringenin (red), ciprofloxacin (green) complexes **(a)** Radius of gyration and **(b)** Solvent accessible surface area.

**FIGURE 8:**
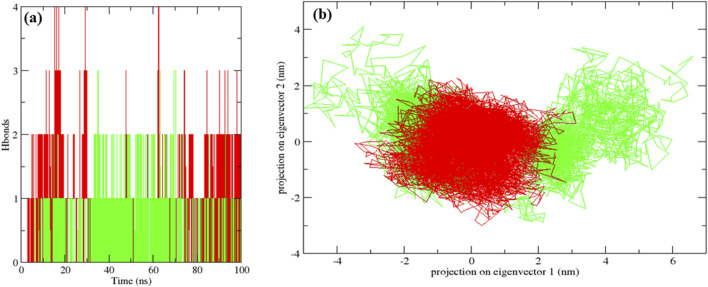
Molecular dynamic simulation results for a duration of 100 ns UDP-N-acetylglucosamine transferase (MurG) native protein (black), 6-Prenylnaringenin (red), ciprofloxacin (green) complexes **(a)** Hydrogen bonds and **(b)** Principle component analysis.

The native MurG protein, the MurG-6-prenylnaringenin complex, and the MurG-ciprofloxacin complex was subjected to 100 ns molecular dynamics simulations. Structural stability was first assessed using root mean square deviation (RMSD), where lower values indicate higher stability (Azmal et al., 2024). All systems remained stable, with the 6-prenylnaringenin complex and the native protein displaying greater stability than the ciprofloxacin complex (Figure 6a). Residue-level flexibility was examined through root mean square fluctuation (RMSF). Fluctuations below 7 Å are generally considered stable (Fatriansyah et al., 2022), and in this case, most residues exhibited low variability, apart from minor fluctuations between residues 70–77 in all systems and additional fluctuation between residues 45–52 in the ciprofloxacin complex (Figure 6b). Compactness of the protein structures was evaluated using the radius of gyration (Rg), where lower values indicate tighter folding (Valathoor and Venugopal, 2024b). The average Rg values were 2.19 nm for the native protein, 2.16 nm for the 6-prenylnaringenin complex, and 2.18 nm for the ciprofloxacin complex, suggesting that the polyphenol complex was slightly more compact (Figure 7a). Solvent-accessible surface area (SASA) analysis showed mean values of 163 nm^2^ for the native protein, 162 nm^2^ for the 6-prenylnaringenin complex, and 164 nm^2^ for the ciprofloxacin complex. The lower SASA of the polyphenol-bound system indicates reduced solvent exposure compared with the other systems (Valathoor and Venugopal, 2024b) (Figure 7b). Hydrogen bond analysis, an indicator of interaction strength (Sapir and Harries, 2017), revealed that the 6-prenylnaringenin complex formed up to four hydrogen bonds, compared with a maximum of three for ciprofloxacin (Figure 8a). Principal component analysis (PCA) was further used to examine conformational dynamics. A more condensed cluster in the PCA plot reflects a reduced conformational space, whereas a dispersed cluster indicates broader sampling (Valathoor and Venugopal, 2024c). The PCA results confirmed that the 6-prenylnaringenin complex was more compact than both the ciprofloxacin and native systems (Figure 8b).

### Molecular mechanics generalized born surface area analysis

**FIGURE 9:**
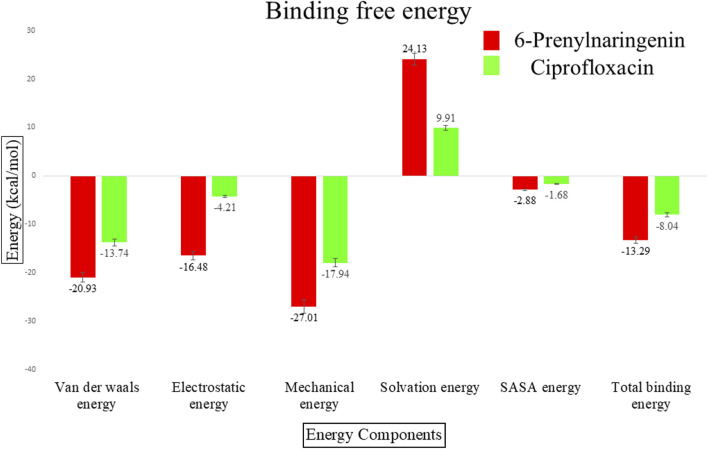
Binding free energy of 6-Prenylnaringenin complex (red) and ciprofloxacin complex (green).

Molecular mechanics generalized Born surface area (MM-GBSA) calculations were carried out to estimate the binding free energies of the MurG-ligand complexes. The MurG-6-prenylnaringenin complex exhibited stronger interactions, with van der Waals, electrostatic, and mechanical energies of −20.93, −16.48, and −27.01 kcal/mol, respectively. In contrast, the MurG-ciprofloxacin complex showed comparatively weaker interactions, with corresponding values of −13.74, −4.21, and −17.94 kcal/mol. Solvation energies were 24.13 kcal/mol for the polyphenol complex and 9.91 kcal/mol for ciprofloxacin, while SASA contributions were −2.88 and −1.68 kcal/mol, respectively. The total binding free energy was calculated as −13.29 kcal/mol for the 6-prenylnaringenin complex and −8.04 kcal/mol for the ciprofloxacin complex, confirming the superior binding strength and stability of the polyphenol ligand (Table 3; Figure 9).

**TABLE 3:** Total binding free energy of MurG with ligand complex through MMGBSA analysis.

S.No	Ligands	Van der waals energy (kcal/mol)	Electrostatic energy (kcal/mol)	Mechanical energy (kcal/mol)	Solvation energy (kcal/mol)	SASA energy (kcal/mol)	Total binding energy (kcal/mol)
1	6-prenylnaringenin	−20.93 ± 1.05	−16.48 ± 1.12	−27.01 ± 1.07	24.13 ± 1.07	−2.88 ± 0.09	−13.29 ± 1.90
2	Ciprofloxacin	−13.74 ± 2.28	−4.21 ± 2.07	−17.94 ± 2.21	9.91 ± 0.59	−1.68 ± 0.01	−8.04 ± 2.28

## Discussion

The rapid rise of antimicrobial and multidrug resistant strains underscores the urgent need for novel antibiotics or alternative therapeutic targets. In this study, UDP N-acetylglucosamine transferase (MurG) was identified as a promising druggable target in *S. typhimurium* through a subtractive genomic approach. Comparative pathway analysis between *H. sapiens* (367 pathways) and *S. typhimurium* (137 pathways) revealed 45 pathogen-specific pathways comprising 620 proteins. Of these, 123 homologous proteins were excluded, and the remaining 497 non-homologous proteins were screened for essentiality. Ten essential proteins were retained, all of which displayed druggable potential: five were cytoplasmic, four were associated with the cytoplasmic membrane, and one was periplasmic. Network analysis highlighted MurG as a central hub protein, making it the final candidate. MurG plays a key role in peptidoglycan biosynthesis, and its inhibition could disrupt cell wall formation in *S. typhimurium*.

Among the key enzymes involved in peptidoglycan biosynthesis, several, including MurA, MurB, MurC, MurD, and MurE, have been extensively studied and targeted in antimicrobial development due to their essential role in bacterial cell wall formation (Subramani and Venugopal, 2025). In contrast, UDP-N-acetylglucosamine transferase (MurG), which catalyzes the final glycosyltransferase step to produce lipid II, remains comparatively underexplored as a therapeutic target in *Salmonella* species (Garde et al., 2021). Its unique enzymatic function and central position within the peptidoglycan biosynthesis pathway enhance its potential as a novel druggable target, further supported by its identification as a hub protein in the present protein-protein interaction network (Subramani and Venugopal, 2025).

The three-dimensional structure of MurG was generated using SWISS-MODEL and validated by Ramachandran plot, ERRAT score, and Z-score analyses, confirming the model’s reliability. Docking experiments with food-derived polyphenols revealed that 6-prenylnaringenin exhibited stronger binding affinity and more favourable hydrogen bond interactions with MurG compared to ciprofloxacin. Molecular dynamics simulations further supported this finding: RMSD and RMSF values indicated stable conformations for the native protein and both complexes, with the 6-prenylnaringenin complex showing greater stability than the ciprofloxacin complex. Radius of gyration (Rg) analysis demonstrated tighter folding of the polyphenol complex, while hydrogen bond analysis revealed a greater number of stable hydrogen bonds. Solvent accessibility calculations showed reduced exposure of the polyphenol complex to surface solvent compared to the native protein and ciprofloxacin complex. Principal component analysis (PCA) indicated that the 6-prenylnaringenin complex occupied a smaller conformational space, reflecting enhanced structural compactness.

Binding free energy (ΔG) calculations reinforced these results, as the MurG-6-prenylnaringenin complex demonstrated stronger overall binding than the MurG-ciprofloxacin complex. The superior stability observed for the MurG-6-prenylnaringenin complex can be attributed to its distinct structural features. Mechanistically, the flavanone core and the flexible prenyl group of 6-prenylnaringenin appear to facilitate a more optimal fit within the binding pocket of the enzyme. The molecular dynamics simulations suggest that this binding induces a more compact and conformationally stable state in the MurG protein. This stability is likely achieved through a robust network of interactions, including strong hydrogen bonds with key residues like Arg162 and extensive hydrophobic contacts involving the prenyl tail. By anchoring the ligand so effectively, these interactions may lock the enzyme in an inactive state, preventing the catalytic conformational changes required for Lipid II synthesis. This provides a clear mechanistic basis for its potential inhibitory function, which contrasts with the less stable and more transient interaction observed with ciprofloxacin.

Taken together, these findings suggest that 6-prenylnaringenin may act as a potent inhibitor of MurG, offering a potential therapeutic strategy against *S. typhimurium*. Similar inhibitory activity has previously been reported for the flavonoid fisetin against the LpoB protein of *S. typhimurium*, supporting the broader antibacterial potential of dietary polyphenols (Valathoor and Venugopal, 2024b). The identification of 6-prenylnaringenin as a direct inhibitor of MurG contributes to the growing body of research on flavonoids as antibacterial agents. For instance, recent work by Zhong et al. (2023) demonstrated a different, synergistic mechanism whereby flavonoids disrupt bacterial iron homeostasis to potentiate the effects of existing antibiotics. This contrasts with the present study, which identifies a direct inhibitory mechanism against an essential enzyme, highlighting the diverse therapeutic strategies offered by this class of natural compounds.

## Conclusion

This work highlights UDP-N-acetylglucosamine transferase (MurG) as a novel and promising therapeutic target in *S. typhimurium*, identified through a systematic subtractive genomic strategy. Structural modelling and computational analyses, including molecular docking and molecular dynamics simulations, demonstrated that the dietary polyphenol 6-prenylnaringenin binds MurG with greater stability and affinity than the widely used antibiotic ciprofloxacin. These findings suggest that 6-prenylnaringenin may serve as a potential lead compound for the development of new anti-*Salmonella* therapeutics. Nevertheless, as this study is restricted to *in silico* investigations, experimental validation through *in vitro* assays and *in vivo* infection models will be essential to confirm its inhibitory potential and therapeutic relevance. Building on these results, future studies could explore structural optimization of 6-prenylnaringenin and its derivatives to enhance binding specificity and efficacy, assess cytotoxicity and pharmacokinetic properties, and investigate potential synergistic effects with existing antibiotics to address multidrug-resistant *Salmonella* infections. Furthermore, future iterations of this discovery pipeline could be significantly enhanced by incorporating advanced machine learning models. For instance, developing a Heterogeneous Information Network (HIN) could accelerate the discovery process by predicting novel drug-target interactions or suggesting candidates for drug repositioning, similar to frameworks recently used in bioinformatics (Zhao et al., 2022; Zhao et al., 2024). Additionally, sequence-based deep learning models could be trained to rapidly screen new compounds for inhibitory potential against MurG, offering predictive power from sequence data alone (Li et al., 2025). Integrating these specific AI-driven strategies represents a promising direction for refining and accelerating the search for novel antimicrobial therapeutics.

## Data Availability

The raw data supporting the conclusions of this article will be made available by the authors, without undue reservation.
